# Synthesis, *in vitro* and *in vivo* evaluation of 3β-[^18^F]fluorocholic acid for the detection of drug-induced cholestasis in mice

**DOI:** 10.1371/journal.pone.0173529

**Published:** 2017-03-08

**Authors:** Stef De Lombaerde, Sara Neyt, Ken Kersemans, Jeroen Verhoeven, Lindsey Devisscher, Hans Van Vlierberghe, Christian Vanhove, Filip De Vos

**Affiliations:** 1 Faculty of Pharmaceutical Sciences, Laboratory of Radiopharmacy, Ghent University, Ghent, Belgium; 2 Ghent University Hospital, Nuclear Medicine, Ghent, Belgium; 3 Ghent University Hospital, Gastroenterology and Hepatology, Ghent, Belgium; 4 IBiTech-MEDISIP-INFINITY, Ghent University, Ghent, Belgium; University of Cambridge, UNITED KINGDOM

## Abstract

**Introduction:**

Drug-induced cholestasis is a liver disorder that might be caused by interference of drugs with the hepatobiliary bile acid transporters. It is important to identify this interference early on in drug development. In this work, *Positron Emission Tomography* (PET)-imaging with a ^18^F labeled bile acid analogue was introduced to detect disturbed hepatobiliary transport of bile acids.

**Methods:**

3β-[^18^F]fluorocholic acid ([^18^F]FCA) was prepared by nucleophilic substitution of a mesylated precursor with [^18^F]fluoride, followed by deprotection with sodium hydroxide. Transport of [^18^F]FCA was assessed *in vitro* using CHO-NTCP, HEK-OATP1B1, HEK-OATP1B3 transfected cells and BSEP & MRP2 membrane vesicles. Investigation of [^18^F]FCA metabolites was performed with primary mouse hepatocytes. Hepatobiliary transport of [^18^F]FCA was evaluated *in vivo* in wild-type, rifampicin and bosentan pretreated FVB-mice by dynamic μPET scanning.

**Results:**

Radiosynthesis of [^18^F]FCA was achieved in a moderate radiochemical yield (8.11 ± 1.94%; non-decay corrected; n = 10) and high radiochemical purity (>99%). FCA was transported by the basolateral bile acid uptake transporters NTCP, OATP1B1 and OATP1B3. For canalicular efflux, BSEP and MRP2 are the relevant bile acid transporters. [^18^F]FCA was found to be metabolically stable. *In vivo*, [^18^F]FCA showed fast hepatic uptake (4.5 ± 0.5 min to reach 71.8 ± 1.2% maximum % ID) and subsequent efflux to the gallbladder and intestines (93.3 ± 6.0% ID after 1 hour). Hepatobiliary transport of [^18^F]FCA was significantly inhibited by both rifampicin and bosentan.

**Conclusion:**

A ^18^F labeled bile acid analogue, [^18^F]FCA, has been developed that shows transport by NTCP, OATP, MRP2 and BSEP. [^18^F]FCA can be used as a probe to monitor disturbed hepatobiliary transport *in vivo* and accumulation of bile acids in blood and liver during drug development.

## Introduction

The production of bile is an important function of the liver. One of the primary constituents are bile acids: amphiphilic molecules synthesized by hepatocytes that play a vital role in digestion of lipids and uptake of fat-soluble vitamins [1]. Bile acids are excreted in the canals of Hering, stored in the gallbladder and excreted into the duodenum via the common bile duct. Bile acids are part of the enterohepatic recirculation and are reabsorbed from the small intestine into the portal vein and transported back to the hepatocytes, where uptake occurs primarily by the basolateral transport protein Na^+^-dependent Taurocholate Cotransporting Polypeptide (NTCP). However, the Organic Anion Transporting Polypeptide (OATP) is also capable of transporting bile acids into the hepatocyte. Hepatic efflux of bile acids towards the bile canaliculi is mediated mainly by the Bile Salt Export Pump (BSEP) and also by the Multidrug Resistance-associated Protein 2 (MRP2) [1].

Drug-induced liver injury (DILI) is an acquired liver disorder responsible for a significant amount of hospitalizations and a prime cause of rejecting new drug candidates during drug development [2,3]. A major part of DILI is represented by drug-induced cholestasis (DIC), which results from inhibition of the bile acid transporters by drugs, leading to a toxic accumulation of bile acids in the liver [4,5].

It is important to detect drug-induced cholestasis early on in drug development. In this regard, nuclear imaging is a powerful tool to investigate interference with the bile acid transporters on a molecular level [6]. Various radiotracers have already been developed that show hepatobiliary transport by these transporters. *Single Photon Emission Computed Tomography* (SPECT)-tracers such as ^99m^Tc Mebrofenin, [^99m^Tc]-DTPA-CDCA and [^99m^Tc]-DTPA-CA are substrates of OATP1B1, OATP1B3 and MRP2 [7,8]. Although the latter two are bile acid analogues, no transport by NTCP or BSEP was observed. [^11^C]dehydropravastatin, [^11^C]rosuvastatin, [^11^C]TIC-Me, [^11^C]glyburide and [^11^C]telmisartan are *Positron Emission Tomography* (PET)-tracers that provide insight into (altered) transport function by OATP, NTCP or MRP2 [9–13]. However, in order to study bile acid transport and the corresponding disturbances, the desired tracer would be a radiolabeled bile acid, predominantly transported by NTCP, BSEP, and by OATP and MRP2 [14].

The synthesis and *in vivo* evaluation of different ^11^C labeled bile acid analogues such as [^11^C]cholylsarcosine was described [15,16]. Although the results are promising, the half-life of the ^11^C-isotope can limit its use. Consequently, a ^18^F labeled bile acid was developed and evaluated *in vivo* in mice by Jia et al. [17]. In this study the ^18^F isotope was incorporated in the bile acid by modification of the carboxyl functional group. Due to this major structural modification however, questions were raised whether the transport mechanism of this tracer was still comparable to endogenous bile acids [18]. To our knowledge, none of these PET bile acid analogues has had its transport characterized *in vitro*.

Therefore, the aim of this work was to develop and evaluate a ^18^F labeled bile acid with minor modifications on the endogenous bile acid structure. This tracer can represent endogenous bile acid transport and can be used as imaging probe for preclinical evaluation of drug interference with the hepatic bile acid transporters. *In vitro* assays were performed to determine the involved bile acid transporters for uptake in, -and efflux out of, the liver. As proof of this concept, imaging experiments in mice were performed with the hepatotoxic drugs rifampicin and bosentan. Rifampicin is a known inhibitor of human/rodent OATP/oatp and MRP2/mrp2 [9,19,20]; bosentan of NTCP/ntcp, and BSEP/bsep [21–23].

## Materials and methods

### Chemicals

All chemicals were at least reagent grade and obtained from Sigma Aldrich (Bornem, Belgium). Cryptand-2.2.2. was purchased at Acros Organics (Geel, Belgium). Cholic acid methyl ester was acquired from TCI Europe (Zwijndrecht, Belgium). All solvents were obtained from Chemlab (Zedelgem, Belgium) and were at least of HPLC-grade. [^3^H]taurocholate and [^3^H]estradiol-17β-glucuronide were obtained from Perkin Elmer. Synthesis of the precursor for radiosynthesis, 3α-Mesyl-7α,12α-Diacetoxy-5β-Cholanic acid methyl ester (MsAcCAME), and general synthesis information can be found in the supplementary data (S1 File).

### Radiosynthesis of 3β-[^18^F]Fluorocholic Acid ([^18^F]FCA)

[^18^F]Fluoride was produced by a ^18^O(p,n)^18^F nuclear reaction with a Cyclone 18/9 cyclotron (IBA). [^18^F]Fluoride (1.5 GBq) was trapped on a Sep-Pak QMA cartridge (Waters, Zellik, Belgium; preconditioned with 5 mL 0.01 M K_2_CO_3_ and 5 mL ultrapure water) and then eluted in a vial with 1 mL cryptand-2.2.2./K_2_CO_3_ solution (0.9 mL acetonitrile (AcN) and 0.1 mL H_2_O containing 20 mg cryptand-2.2.2. and 2 mg K_2_CO_3_). The solution was azeotropically evaporated to dryness under a gentle nitrogen flow at 100°C, followed by two additional drying steps with 500 μL AcN.

**Fig 1:**
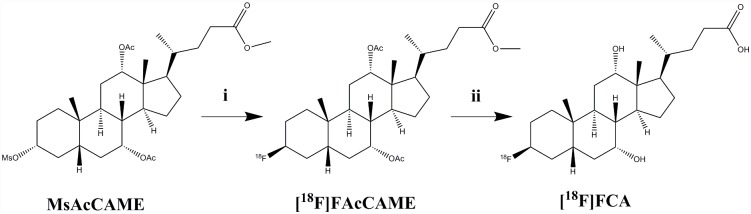
Radiosynthesis of 3β-[^18^F]Fluoro-7α,12α-Diacetoxy-5β-Cholanic Acid Methyl Ester ([^18^F]FAcCAME) and subsequent deprotection to 3β-[^18^F]Fluorocholic Acid ([^18^F]FCA). *i*: [^18^F]Fluoride/K_222_-K_2_CO_3_; DMSO; 120°C; 20 min. *ii*: 1 M NaOH; 120°C; 10 min.

The precursor for radiosynthesis (Fig 1; 4 mg MsAcCAME dissolved in 200 μL anhydrous DMSO) was added to the vial. The reaction mixture was heated for 20 minutes at 120°C. Radiofluorination of precursor molecule MsAcCAME was monitored by TLC (10 mM NH_4_Ac:AcN 1:4 v:v). The vial was subsequently cooled to room temperature and NaOH (100 μL 3 M) was added to allow deprotection of the labeled intermediate [^18^F]FAcCAME. Deprotection took place for 10 minutes at 120°C. The vial was cooled to room temperature and the reaction mixture was purified by means of semi-preparative HPLC (Grace Econosphere C18 10.0x250 mm, 10 μm; 6 mL/min AcN:H_2_O 10:90 v:v ->AcN 100% in 20 minutes as mobile phase; radiodetection (Ludlum Measurements Inc)). The desired HPLC-fraction was collected, diluted with ultrapure water and loaded onto a Sep-Pak C18 cartridge (preconditioned with 5 mL EtOH and 5 mL ultrapure water). The cartridge was washed with 5 mL ultrapure water and eluted with 500 μL EtOH. The solvent was evaporated under a gentle nitrogen flow and [^18^F]FCA was reformulated with 500 μL phosphate buffered saline (PBS) pH 7.4. This formulation was subjected to quality control by means of analytical HPLC with radio-and UV detection (205 nm; Waters) using a Grace Alltima C18 (4.6x250 mm; 5 μm) column and 1 mL/min H_2_O:AcN 70:30 v:v as mobile phase. The retention time of [^18^F]FCA was compared to that of a non-radioactive reference compound.

1 MBq of [^18^F]FCA was used to spike an octanol/PBS 50:50 v:v pH 7.4 mixture in a test tube. After mixing and centrifugation (5 min; 1100 g), aliquots were taken from each layer and radioactivity was measured in a NaI (Tl) scintillation counter (Capintec; Ramsey, NJ, USA). The LogD value of [^18^F]FCA was calculated as the logarithm of the ratio of counts in octanol and PBS.

### *In vitro* characterization

#### Cell culture

Transport of 3β-fluorocholic acid by NTCP, OATP1B1 and OATP1B3 was assessed using stable transfected cell lines. Chinese hamster ovary (CHO) cells, Human Embryonic Kidney (HEK), HEK293-OATP1B3 cells were purchased from Solvo Biotechnologies; CHO-NTCP and HEK293-OATP1B1 cells were a kind gift from the same company. CHO cells were cultured in Dulbecco’s modified Eagle’s medium (DMEM)/F12 with 10% fetal calf serum, 0.3 mM L-proline, 2 mM glutamine and penicilline/streptomycine (50 U/mL). HEK cells expressing OATP1B1, OATP1B3 and control cell lines were cultured in DMEM glutamax (high glucose/pyruvate) with 10% fetal calf serum, MEM non-essential amino acid supplement and penicilline/streptomycine (50 U/mL). All cell lines were cultured in a 37°C 5% CO_2_ environment.

#### *In vitro* uptake assay

Tritium labeled model substrates were used as positive controls for transporter expression: [^3^H]taurocholate ([^3^H]TC) for NTCP; [^3^H]estradiol-17β-glucuronide ([^3^H]EbG) for OATP1B1 and OATP1B3. A kinetic profile of the uptake of these model compounds was determined and can be found in S2 File. Transport of FCA by the NTCP, OATP1B1 and OATP1B3 transporters was assessed by inhibition of the tritium labeled model substrates uptake.

Cells were seeded in 24-well plates at 400,000 cells per well. After 24 hours, the culture medium was removed and cells were washed twice with 1 mL 10 mM HEPES-Hank’s Balanced Salt Solution (HBSS; with Ca and Mg; 37°C pH 7.4) washing buffer. Dosing solutions were prepared by supplementing the washing buffer to obtain 1 μM [^3^H]TC for CHO cells, 1 μM [^3^H]EbG for HEK cells and varying concentrations of cold FCA (0–1000 μM). Of the dosing solution, 250 μL was added to the washed cells and incubation took place at 37°C for 15 minutes. The plates were cooled on ice and 1 mL 1% BSA ice-cold HBSS (with Ca and Mg) was added. Cells were washed twice with 2 mL ice-cold HBSS (with Ca and Mg) and were finally lysed with 250 μL 0.1 M NaOH. The plates were shaken for 15 minutes at 37°C. Of the lysate, 150 μL was used for liquid scintillation counting (TriCarb 2900 TR; Perkin Elmer) and 25 μL was subjected to a BCA assay (ThermoFisher Scientific) to determine protein content. Data points were collected in triplicate. IC50-values were calculated with Graphpad Prism v3.00 (Graphpad software).

#### *In vitro* efflux assay

Transport of 3β-fluorocholic acid (FCA) by the bile acid efflux pumps BSEP and MRP2 was determined by a competition assay with [^3^H]TC and [^3^H]EbG in BSEP and MRP2 membrane vesicles (Pharmtox). The assay was performed in 96-well plates. Each well contained 3.75 μg membrane vesicle, 10 mM MgCl_2_, 4 mM ATP, 10 mM Tris, 250 mM sucrose, 0–10 mM FCA and 2 μM [^3^H]TC or [^3^H]EbG. The final volume in each well was 30 μL. The plates were incubated for 10 minutes at 37°C and uptake was halted by the addition of 150 μL ice-cold buffer solution. The contents of each well were transferred to a glass fiber filter plate (Multiscreen HTS plates; Merck Millipore) and washed 3 times with 200 μL of ice-cold buffer solution. Vesicles were lysed by incubation with 100 μL 0.1 M NaOH for 10 minutes at room temperature. An 80 μL aliquot of this solution was used for liquid scintillation counting. Data points were collected in triplicate. IC50-values were calculated with Graphpad Prism v3.00.

### *In vitro* investigation of [^18^F]FCA stability

The stability or metabolization of [^18^F]FCA was assessed in its formulation, in mouse serum and in presence of primary mouse hepatocytes. Mouse serum (Sigma Aldrich, Bornem, Belgium) was spiked with 37 MBq [^18^F]FCA (1.0 mL total volume) and incubated at 37°C for 5, 10, 30 and 60 minutes.

Primary mouse hepatocytes were isolated as described previously [24], formulated in DMEM medium at 1 million cells/mL and immediately incubated with 37 MBq [^18^F]FCA (1.0 mL total volume) at 37°C, 5% CO_2_ for 5, 10, 30 and 60 minutes. A control sample without hepatocytes was included.

At the indicated timepoints, 100 μL serum or hepatocyte incubation medium was withdrawn and 100 μL AcN was added. Samples were then centrifuged at 13000 g for 5 minutes and the supernatant was analyzed by the same semi-preparative RP-HPLC method as for the radiosynthesis of [^18^F]FCA.

### *In vivo* evaluation of [^18^F]FCA

Hepatobiliary transport of the tracer was evaluated in female wild-type FVB mice (5 weeks old, Charles River). The animals were housed in accordance with European Ethics Committee guidelines. The animal studies were approved by the Animal Experimental Ethical Committee of Ghent University (ECD 15/69). Food and water was provided *ad libitum*. Animals were fasted overnight before the PET-scan.

Mice were anesthetized with 1.5 v:v % isoflurane in 100% O_2_ for the duration of the experiment. An intravenous polyethylene line was placed in the lateral tail vein. The animals were placed in the small animal PET/CT-scanner (FLEX Triumph II small animal PET/CT-scanner; PET field of view: 7.5 cm axial; 1.3 mm spatial resolution; TriFoil Imaging) on a heated bed. A CT-scan was acquired for anatomical correlation.

*In vivo* characteristics of [^18^F]FCA were investigated by a two hour dynamic PET scan with 30 MBq [^18^F]FCA, injected intravenously (n = 3). Four and six hours post-injection, a 30 minute static scan was performed.

The interaction of rifampicin (Sigma Aldrich) and bosentan (Activate Scientific) on *in vivo* [^18^F]FCA transport was examined. FVB-mice were divided into 3 different groups (n = 3 per group): rifampicin, bosentan, and a control DMSO vehicle treated group). Both drugs were administered in doses of 100 mg/kg, intra-peritoneal in DMSO:PBS 70:30 v:v (rifampicin) or DMSO (bosentan) 1 hour before the start of the PET-scan. An additional dose (25 mg/kg for rifampicin in DMSO:PBS 40:60 v:v, 50 mg/kg for bosentan in DMSO) was co-injected intravenously with the tracer.

For each scan, 10 MBq of [^18^F]FCA was administered intravenously and a 1 hour dynamic scan was started. Immediately after the dynamic scan, 18 MBq [^18^F]FDG was injected intravenously. After 20 minutes, a 20 minutes static scan was taken.

PET-data were obtained in list-mode and were iteratively reconstructed (50 iterations) in frames of 15 seconds for the first 10 minutes, followed by 50 frames of 1 minute. ROI’s were drawn manually over liver, gallbladder and intestines using PMod software. A ROI was drawn in the left ventricle on the static [^18^F]FDG scan to obtain an image-derived arterial blood input curve [25]. The uptake of [^18^F]FCA in arterial blood, liver, gallbladder and intestines was expressed as a % injected dose (% ID) and normalized for the weight of a 20 gram mouse. Time-activity curves (TAC’s) were generated using these values.

AUC, maximum % ID, and time to peak values were determined for the TAC’s with Graphpad Prism v3.00. Differences between 2 groups were determined with the non-parametric Mann-Whitney U test. P-values ≤ 0.05 were considered significant.

## Results

### Radiosynthesis of 3β-[^18^F]Fluorocholic Acid ([^18^F]FCA)

Precursor molecule MsAcCAME was labeled with [^18^F]fluoride with a 30% radiofluorination yield. The radiolabeled intermediate [^18^F]FAcCAME was converted to [^18^F]FCA in the subsequent deprotection step. Heating to at least 100°C was necessary, as no significant deprotection was observed at low temperatures.

The radiosynthesis of [^18^F]FCA was achieved in 100 minutes with 8.11 ± 1.94% non-decay corrected radiochemical yield (mean ± SD; n = 10). Radiochemical purity was assessed by analytical HPLC (T_r_ [^18^F]FCA = 3.3 min) and was >99%. The identity of [^18^F]FCA was confirmed by coinjection of cold reference compound 3β-fluorocholic acid. The logD value in octanol/PBS pH 7.4 was 1.19 ± 0.10 (n = 3).

### *In vitro* uptake

**Fig 2:**
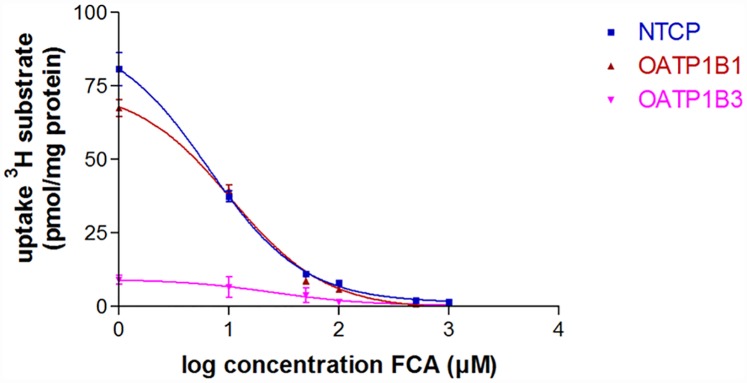
FCA concentration-dependent inhibition of [^3^H]Taurocholate ([^3^H]TC) and [^3^H]Estradiol-17β-Glucuronide ([^3^H]EbG) uptake in NTCP and OATP expressing cell lines respectively. Values are expressed as mean ± SD (n = 3).

To determine the transport characteristics of 3β-fluorocholic acid, a competition experiment with ^3^H-labeled substrates of NTCP, OATP1B1 and OATP1B3 was performed. Uptake of [^3^H]TC and [^3^H]EbG in control and transfected cell lines can be found in S2 File. After 15 minutes of incubation without added FCA, [^3^H]TC uptake in CHO-NTCP cells was 95.2 ± 6.0 pmol/mg protein; [^3^H]EbG uptake in HEK-OATP1B1 and OATP1B3 was 84.0 ± 7.7 pmol/mg protein and 8.6 ± 1.3 pmol/mg protein respectively. Uptake of [^3^H]TCA NTCP and [^3^H]EbG was inhibited by FCA in a concentration dependent manner (Fig 2). IC50 values for this competition assay were 6.6 ± 0.8 μM for NTCP; 10.5 ± 1.1 μM for OATP1B1 and 25.2 ± 12.7 μM for OATP1B3.

### *In vitro* efflux

**Fig 3:**
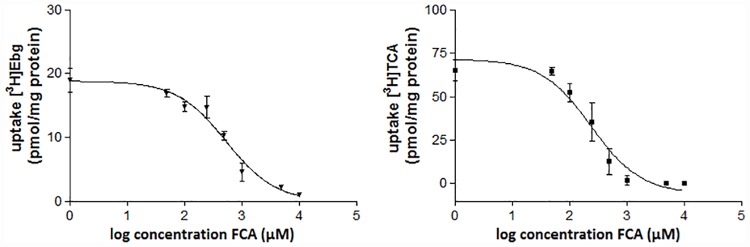
FCA concentration dependent inhibition of [^3^H]Taurocholate ([^3^H]TC) and [^3^H]Estradiol-17β-Glucuronide ([^3^H]EbG) in BSEP and MRP2 vesicles respectively. Values are expressed as mean ± SD (n = 3).

Transport of FCA by the bile salt transporters BSEP and MRP2 was assessed by a competition assay with [^3^H]TCA and [^3^H]EbG. After 10 minutes of incubation with [^3^H]TCA, uptake in BSEP vesicles was 65.2 ± 6.2 pmol/mg protein; [^3^H]EbG uptake in MRP2 vesicles was 18.5 ± 3.8 pmol/mg protein. Uptake of these model substrates was inhibited by FCA in a concentration dependent manner (Fig 3). IC50 values of this inhibition were 252.6 ± 76.0 μM for BSEP and 577.2 ± 300.2 μM for MRP2.

### *In vitro* investigation of [^18^F]FCA stability

[^18^F]FCA was found to be stable in its PBS formulation for at least 6 hours at room temperature. No degradation or metabolization products of [^18^F]FCA were detected at any examined time point in the presence of mouse serum or primary mouse hepatocytes (data in S3 File).

### *In vivo* evaluation of [^18^F]FCA

**Fig 4:**
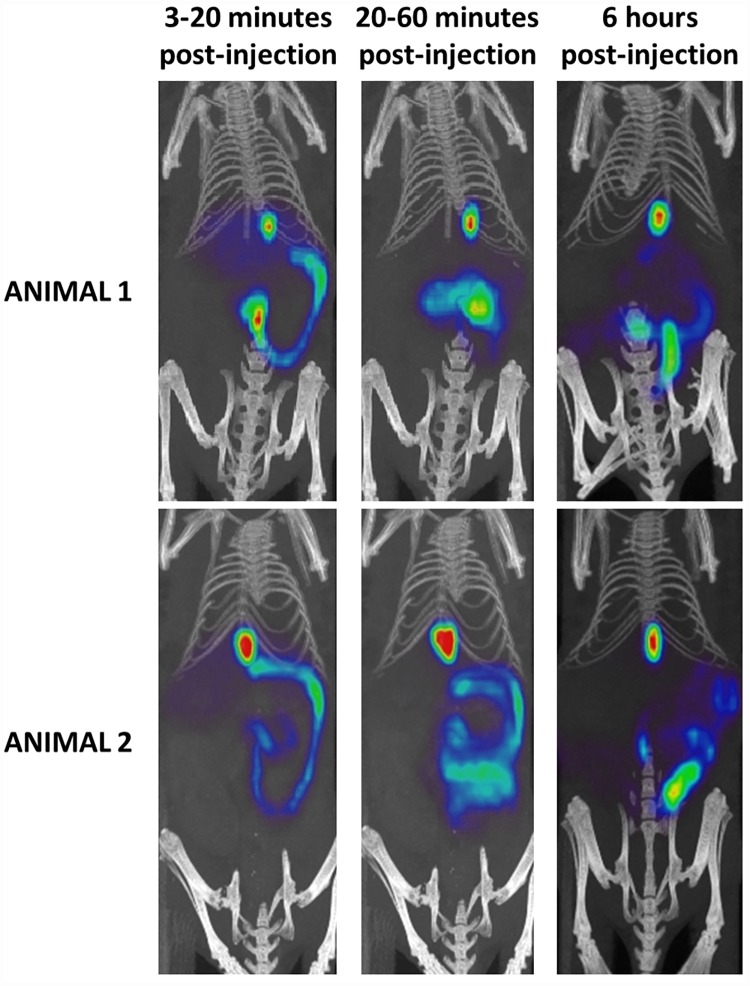
Maximum intensity projection PET/CT images of [^18^F]FCA in 2 wild-type FVB-mice at different time points. Images were generated in AMIDE Medical Image Data Examiner software; slice thickness: 12 mm. In the early phase (3–20 minutes post-injection), the liver, gallbladder and intestines are visible. In a later phase (20–60 minutes post-injection), all radioactivity is excreted from the liver to the gallbladder and intestines. The late phase PET/CT images (6 hours post-injection) show that [^18^F]FCA remains present only in gallbladder and intestines: visual analysis of all images showed no uptake in other organs than liver, gallbladder and intestines.

**Fig 5:**
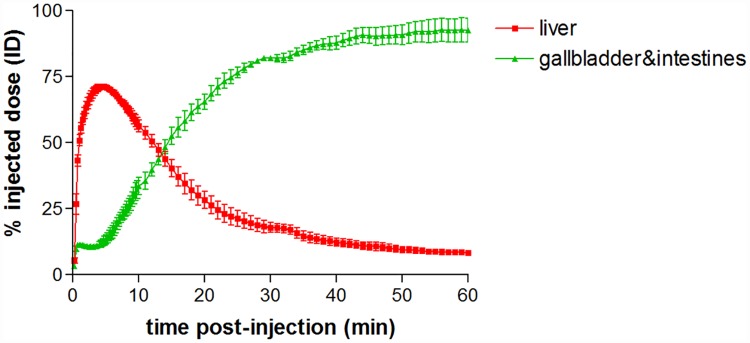
Time-activity curves of [^18^F]FCA in liver and gallbladder & intestines of wild-type FVB-mice (n = 3). Uptake of [^18^F]FCA is expressed as % injected dose and normalized for a 20 g mouse. Values are expressed as mean ± SD.

Fig 4 shows the PET/CT images of [^18^F]FCA in wild-type FVB-mice at different time points. The accumulation of the tracer in the liver reached a maximum % ID of 71.8 ± 1.2% ID with the time to peak being 4.5 ± 0.5 min, after which a decrease to baseline levels was observed, indicating excretion of radioactivity. A corresponding increase of [^18^F]FCA was observed in the gallbladder and intestines. After 1 hour, almost all radioactivity was found in the gallbladder and intestines (93.3 ± 6.0% ID) (Fig 5, the corresponding metrics are shown in Table 1). No uptake was observed in the urinary bladder or other organs.

**Table 1:** Metrics of [^18^F]FCA in liver and gallbladder & intestines in wild-type FVB-mice (n = 3). Values are expressed as mean ± SD.

	Wild-type
**Max % injected dose liver**	71.8 ± 1.2
**Time to peak liver(min)**	4.5 ± 0.5
**AUC gallbladder & intestines % injected dose(% ID.min)**	4105 ± 36

**Fig 6:**
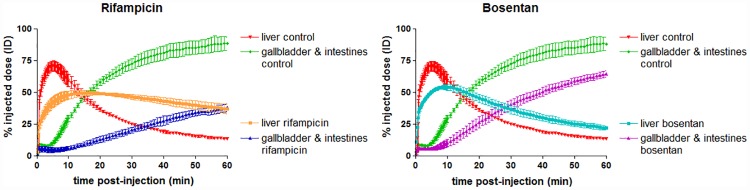
Liver and gallbladder & intestines time-activity curves of [^18^F]FCA in control, rifampicin and bosentan treated FVB-mice (n = 3 per group). Uptake of [^18^F]FCA is expressed as % injected dose and normalized for a 20 g mouse. Values are expressed as mean ± SD.

**Fig 7:**
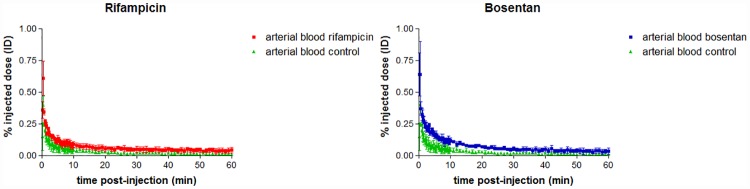
Time-activity curves of [^18^F]FCA in arterial blood for vehicle (green), rifampicin (red) and bosentan (blue) treated mice (n = 3 per group). Uptake of [^18^F]FCA is expressed as % injected dose and normalized for a 20 g mouse. Values are expressed as mean ± SD.

The hepatobiliary transport of [^18^F]FCA was studied in mice who were dosed with rifampicin or bosentan. TAC’s and metrics of [^18^F]FCA distribution in rifampicin and bosentan treated mice are depicted in Fig 6 and Table 2. Rifampicin and bosentan treated mice showed an altered distribution of [^18^F]FCA compared to control animals: time to peak of the liver TAC significantly increased 2.9 and 1.7-fold versus control for rifampicin and bosentan respectively. The maximum % ID in the liver was also significantly lower than control for both compounds (49.7 ± 1.6% and 54.4 ± 1.7% for rifampicin and bosentan respectively, compared to 71.2 ± 3.5% for control). Efflux towards the gallbladder and intestines was impaired: the AUC-value of the gallbladder & intestines TAC was 3.1 times lower than control for rifampicin and 1.7 times lower for bosentan. The AUC of the arterial blood TAC was 2.1-fold and 2.5-fold higher than control for rifampicin and bosentan respectively (see Fig 7).

**Table 2:** Time-activity curve metrics of [^18^F]FCA in control, rifampicin and bosentan treated animals (n = 3 per group). Values are expressed as mean ± SD. *: significant difference compared to control group. A p-value of 0.05 was considered significant.

	Control	Rifampicin 100 mg/kg IP+ 25 mg/kg IV	Bosentan 100 mg/kg IP+ 50 mg/kg IV
**Max % injected dose liver**	71.2 ± 3.5	49.7 ± 1.6*	54.4 ± 1.7*
**Time to peak liver (min)**	5.5 ± 1.1	16.0 ± 5.0*	9.3 ± 1.6*
**AUC gallbladder &intestines % injected dose (% ID.min)**	3718 ± 182	1208 ± 132*	2196 ± 181*
**AUC arterial blood % injected dose (% ID.min)**	1.909 ± 0.572	3.916 ± 0.957*	4.524 ± 0.466*

## Discussion

Bile acids are amphiphilic steroid derivatives that play a vital role in the digestion of lipids and uptake of fat-soluble vitamins. In many species, including rodents and humans, cholic acid makes up the larger part of the bile acid spectrum [26]. Cholic acid is therefore an opportune target to radiolabel and use as an imaging probe for hepatobiliary transport of bile acids in mice and humans alike. In this article, a ^18^F labeled bile acid, 3β-[^18^F]fluorocholic acid, is introduced to assess hepatobiliary transport of bile acids in mice. This ^18^F PET-tracer shows remarkable structural resemblance to cholic acid, differing only in substitution of the 3α-OH with a 3β-fluorine. It was hypothesized that this modification would not have a major effect on transport by the bile acid transporters, as fluorine is isosteric and iso-electronic with a OH-group. Moreover, the 3α-OH group is not an absolute requisite for active bile acid transport [27,28].

The radiofluorination of MsAcCAME with [^18^F]fluoride showed an incorporation yield of 30%, which is in line with radiofluorination of a secondary mesylate directly attached to a cyclohexane ring [29]. This moderate yield can be attributed to the sterically hindered environment of the leaving group. After purification and formulation of [^18^F]FCA in PBS, no radiochemical degradation products, metabolization or radiodefluorination was observed: the tracer remained stable for at least 6 hours in its formulation and at least 1 hour in presence of mouse serum and primary mouse hepatocytes. The logD value of [^18^F]FCA was in accordance with the logD value of cholic acid (logD = 1.1) [30].

*In vitro* transport evaluation of [^18^F]FCA revealed that the hepatic bile acid transporters involved in uptake and efflux are NTCP, OATP1B1, OATP1B3, BSEP and MRP2, which is also the case for endogenous bile acids [1,31,32]. Substitution of the 3α-OH of cholic acid with a 3β-fluorine did not have an effect on the ability to be transported by the same hepatobiliary transporters as endogenous bile acids.

The *in vivo* evaluation of [^18^F]FCA in wild-type FVB mice showed that the tracer is rapidly and exclusively taken up into the liver after intravenous injection (4.5 min to reach 71.8 maximum % ID). Compared to ^99m^Tc-mebrofenin in FVB-mice (2.2 minutes to reach 51.8 maximum % ID) [33], uptake into the liver is slightly slower and reaches a comparable % ID. This behavior is similar to ^11^C labeled bile acid analogues which also show a fast and exclusive hepatic uptake (<7 minutes), in pigs [15,16]. On the other hand, ^99m^Tc labeled bile acids ^99m^Tc CDCA and ^99m^Tc CA reach a lower peak value in the liver at reduced rate (12.7 minutes to reach 37.3 max % ID and 12.0 minutes to reach 25.7 max % ID respectively) [8]. Following rapid uptake into the liver, [^18^F]FCA is excreted into the bile ducts, gallbladder and finally accumulates in the intestines. There was no accumulation of tracer visible in other organs for up to 6 hours post-injection. After 1 hour, activity in the gallbladder and intestines reached a plateau of 93.3% ID. This value is comparable to that of ^99m^Tc mebrofenin (78.1% ID) and higher than the maximal % ID in gallbladder and intestines of the ^99m^Tc labeled bile acids (62.2% ID for ^99m^Tc CDCA; 47.8% for ^99m^Tc CA). Because of their partial urinary clearance, ^99m^Tc bile acids show a lower uptake into the liver and a lower accumulation in gallbladder and intestines.

[^18^F]FCA was developed to detect disturbed hepatobiliary transport of bile acids. As proof of concept, the oatp and mrp2 inhibitor rifampicin and the ntcp and bsep inhibitor bosentan were administered to FVB-mice and subjected to a PET-scan with [^18^F]FCA. Both rifampicin and bosentan caused a significant delay in hepatic uptake and lower maximum % ID in the liver and both drugs gave rise to an increased amount of [^18^F]FCA in arterial blood, which is in line with an observed increase in serum bile acids after administration of these drugs [19,22]. When an identical dose of rifampicin was administered to FVB-mice scanned with ^99m^Tc mebrofenin [33], a complete block of canalicular efflux was observed on the SPECT-scan, whereas [^18^F]FCA excretion into bile is only hindered. This difference in excretion can be attributed to the maximal blocking effect of rifampicin on ^99m^Tc mebrofenin transport, as this tracer is an exclusive oatp and mrp2 substrate [7]. [^18^F]FCA however can still be excreted into bile by bsep.

This observed *in vivo* inhibition confirms our *in vitro* findings that [^18^F]FCA uses OATP and NTCP as the basolateral hepatic uptake systems; for canalicular hepatic efflux, BSEP and MRP2 are the bile acid transporters involved. [^18^F]FCA can therefore be used as a probe for *in vivo* bile acid uptake, hepatobiliary efflux, and related disturbances. This tracer can be a valuable tool to detect drug-induced cholestasis during preclinical evaluation of new drugs *in vivo*.

## Conclusion

A ^18^F labeled cholic acid analogue, 3β-[^18^F]fluorocholic acid ([^18^F]FCA), was synthesized in a moderate radiochemical yield. *In vitro* data shows that [^18^F]FCA is metabolically stable and is taken up into the hepatocyte by the basolateral transporters NTCP, OATP1B1 and OATP1B3. Canalicular efflux is mediated by MRP2 and BSEP. To our knowledge, [^18^F]FCA is the first PET bile acid to undergo *in vitro* characterization. *In vivo*, [^18^F]FCA is rapidly taken up into the liver and excreted in gallbladder and intestines. Significant alterations in [^18^F]FCA uptake and efflux parameters were observed with the hepatotoxic drugs rifampicin and bosentan. [^18^F]FCA shows favorable *in vitro* and *in vivo* characteristics that are comparable to endogenous bile acids and can therefore be a promising imaging probe in preclinical drug development for the detection of drug-induced altered hepatobiliary transport of bile acids.

## Supporting information

S1 File Synthesis of [^18^F]FCA precursor for radiosynthesis and FCA reference compound. (DOCX)

S2 File Kinetic profile of [^3^H]TC and [^3^H]EbG in CHO-NTCP and HEK-OATP1B1/1B3 cells. (DOCX)

S3 File Stability assessment of [^18^F]FCA in mouse serum and in presence of mouse primary hepatocytes. (DOCX)
