# Supplementary material for: Synthesis, in vitro and in vivo evaluation of 3β-[18F]fluorocholic acid for the detection of drug-induced cholestasis in mice
Source: PLoS One. 2017 Mar 8;12(3):e0173529. doi: 10.1371/journal.pone.0173529 (PMC5342262; doi:10.1371/journal.pone.0173529)
Supplement: S1 File — (DOCX) [file pone.0173529.s001.docx]

# Synthesis, *in vitro* and *in vivo* evaluation of 3β-[^18^F]fluorocholic acid for the detection of drug-induced cholestasis in mice.

# Supplementary data

# S1 text: Synthesis of [^18^F]FCA precursor for radiosynthesis and FCA reference compound

## General synthesis information

S1 Fig 1 displays the different synthesis steps to synthesize the precursor for radiosynthesis and the non-radioactive reference compound. TLC analysis during and after each synthesis step was performed on aluminium sheets, coated with silica gel (silica gel 60; Sigma Aldrich, Bornem, Belgium) and a mixture of EtOAc and hexane as mobile phase. Spots were visualized by spraying a 50% phosphoric acid solution on the plates and heating them.

S1 Fig 1: synthesis of [^18^F]FCA precursor for radiosynthesis and FCA reference compound
i: 4-DMAP, pyridine, Ac_2_O, overnight, RT, 100 %; ii: AcCl, MeOH, 24h, 0°C to RT, 75 %; iii: MsCl, pyridine, 6h, 0°C to RT, 88 %; iv: DAST, CH_2_Cl_2_, 0°C to RT, 77 %; v: 1 M NaOH, EtOH, 3h, reflux, 76 %

Purification of the crude reaction products was achieved by silica gel column chromatography (Sigma Aldrich, Bornem, Belgium). A mixture of EtOAc and hexane was used as mobile phase. Fractions were collected and analyzed by aforementioned TLC method to isolate the desired compound.

^1^H NMR spectra were recorded with a Varian Mercury BB 300 MHz (Palo Alto, CA, USA). Mass spectra were obtained by ESI with a Waters LCT-premier XE, TOF high resolution mass spectrometer (Milford, MA, USA).

## Synthesis of precursor molecule for radiolabeling and 3β-fluorocholic acid reference compound

### 3α,7α,12α-Triacetoxy-5β-Cholanic acid methyl ester (2)

Acetyl protecting groups were introduced on the hydroxyl functions of 3α,7α,12α-trihydroxy 5β-cholanic acid methyl ester based on a similar reaction described in literature [1]. To a stirred solution of 3α,7α,12α-trihydroxy 5β-cholanic acid methyl ester (4 g, 9,465 mmol) and 4-DMAP (240 mg, 1.964 mmol) in anhydrous pyridine (30 mL), acetic acid anhydride (28 mL, 296 mmol) was added. The reaction mixture was stirred overnight at room temperature, after which the mixture was poured into water (200 mL) and extracted with EtOAc (3x 200 mL). The organic phase was washed with 1 M HCl (200 mL), brine (200 mL), and water (200 mL). The washed solvent was dried with Na_2_SO_4_ and evaporated. The remaining residue was subjected to column chromatography using EtOAc:hexane (50:50). The desired fractions were combined and evaporated, yielding a white solid (100 %). MS: 566.3677 [M+NH_4_]. ^1^H NMR (DMSO-d_6_; 300 MHz): 4.93 (1H, m); 4.75 (1H, m); 4.42 (1H, m); 3.54 (3H, s); 2.04 (3H, s); 1.97 (3H, s); 1.95 (3H, s).

### 3α-Hydroxy-7α,12α-Diacetoxy-5β-Cholanic acid methyl ester (3)

The partial deprotection of 3α,7α,12α-trihydroxy 5β-cholanic acid methyl ester was based on a similar reaction described in literature [1]. 3α,7α,12α-trihydroxy 5β-cholanic acid methyl ester (2 g; 3.645 mmol) was dissolved in anhydrous MeOH (20 mL). The solution was cooled to 0°C. AcCl (340 µL), dissolved in anhydrous MeOH (3 mL), was slowly added. The mixture was stirred for 24h at room temperature. The reaction was stopped by adding saturated NaHCO_3_ (50 mL) and extracted with EtOAc (3x 100 mL). The organic phase was dried over Na_2_SO_4_, concentrated *in vacuo* and subjected to column chromatography using EtOAc:hexane (1:3). The desired fractions were combined and evaporated, yielding a white solid (75%). MS: 524.3576 [M+NH_4_]. ^1^H NMR (DMSO-d_6_; 300 MHz): 4.92 (1H, m); 4.73 (1H, m); 4.44 (1H; m); 3.54 (3H, s); 3.19 (1H, m); 2.03 (3H, s); 1.97 (3H, s).

### 3α-Mesyl-7α,12α-Diacetoxy-5β-Cholanic acid methyl ester (4)

The introduction of the mesyl leaving group was based on a similar reaction described in literature [2]. 3α-hydroxy-7α,12α-dihydroxy 5β-cholanic acid methyl ester (150 mg, 0.296 mmol) was dissolved in anhydrous pyridine (5 mL). The solution was cooled to 0°C and MsCl (69 µL; 0.888 mmol) was slowly added. The mixture was stirred for 6h at room temperature. Then, the reaction mixture was diluted with 50 mL 6 M HCl in brine and extracted with CH_2_Cl_2_ (3x 50 mL). The organic phase was dried with MgSO_4_ and concentrated *in vacuo*. This residue was purified by column chromatography using EtOAc:hexane (2:3). The desired fractions were combined and evaporated, yielding a white solid (88 %). MS: 602.3380 [M+NH_4_]. ^1^H NMR (DMSO-d_6_; 300 MHz): 4.95 (1H, m); 4.78 (1H, m); 4.41 (1H; m); 3.55 (3H, s); 3.13 (3H; s); 2.05 (3H, s); 1.99 (3H, s).

### 3β-Fluoro-7α,12α-Diacetoxy-5β-Cholanic acid methyl ester (5)

3α-hydroxy-7α,12α-dihydroxy 5β-cholanic acid methyl ester (400 mg; 0.789 mmol) was dissolved in anhydrous CH_2_Cl_2_ (10 mL). The solution was cooled to 0°C and DAST (200 µL; 1.514 mmol) was slowly added. The mixture was stirred for 3h at room temperature and then diluted with CH_2_Cl_2_ (30 mL) and saturated NaHCO_3_ (50 mL). The organic phase was washed with water (3x100 mL), dried with MgSO_4_ and concentrated *in vacuo*. The residue was purified by column chromatography, using EtOAc:hexane (3:7). The desired fractions were combined to yield a white solid (77%). MS: 526.3530 [M+NH_4_]. ^1^H NMR (DMSO-d_6_; 300 MHz): 4.93-4.73 (3H, m); 3.54 (3H, s); 2.02 (3H, s); 1.99 (3H, s).

### 3β-Fluoro-7α,12α-Dihydroxy-5β-Cholanic acid (6)

A solution of 3β-fluoro-7α,12α-dihydroxy 5β-cholanic acid methyl ester (260 mg; 0.511 mmol) in 1 M NaOH and EtOH (20 mL) was refluxed for 3 hours. Then, the reaction mixture was acidified with 1 M HCl and extracted with CH_2_Cl_2_ (3x 50 mL). The organic phase was washed with brine (50 mL), saturated NaHCO_3_ (50 mL), water (50 mL). Finally, the organic phase was dried over MgSO_4_ and evaporated, yielding a white solid (76 %). Purity was assessed by TLC-analysis, and no further purification was performed. MS: 428.3193 [M+NH_4_]. ^1^H NMR (DMSO-d_6_; 300 MHz): 11.98 (1H, s); 4.85-4.69 (1H, d); 4.12 (2x 1H overlap; m) 3.76 (1H, s); 3.60 (1H, s).

## Literature:

[1] F Májer, R Sharma, C Mullins, et al.,“New highly toxic bile acids derived from deoxycholic acid, chenodeoxycholic acid and lithocholic acid,”*Bioorganic Med. Chem.*,vol.22,pp.256–268,2014.

[2] J Rohacova, ML Marin, A Martínez-Romero, et al.,“Synthesis of new, UV-photoactive dansyl derivatives for flow cytometric studies on bile acid uptake.,”*Org.Biomol.Chem.*,vol. 7,pp.4973–80,2009.
