# Supplementary material for: Synthesis, in vitro and in vivo evaluation of 3β-[18F]fluorocholic acid for the detection of drug-induced cholestasis in mice
Source: PLoS One. 2017 Mar 8;12(3):e0173529. doi: 10.1371/journal.pone.0173529 (PMC5342262; doi:10.1371/journal.pone.0173529)
Supplement: S2 File — (DOCX) [file pone.0173529.s002.docx]

# Synthesis, in vitro and in vivo evaluation of 3β-[18F]fluorocholic acid for the detection of drug-induced cholestasis in mice.

# Supplementary data

# S2 file: kinetic profile of [^3^H]TC and [^3^H]EbG in CHO-NTCP and HEK-OATP1B1/1B3

The NTCP transfected CHO cellines showed uptake of [^3^H]TC in a time-dependent manner. After 15 minutes of incubation, the uptake of [^3^H]TC was 101-fold higher in the CHO-NTCP cells than control (135.5 pmol/mg protein vs. 1.33 pmol/mg protein respectively). [^3^H]EbG was taken up in the HEK-OATP1B1 and HEK-OATP1B3 cells, but not in control. After 15 minutes, uptake of [^3^H]EbG was 115 and 13 times higher in HEK-OATP1B1 and HEK-OATP1B3 respectively (163.9 pmol/mg protein for HEK-OATP1B1; 19.0 pmol/mg protein for HEK-OATP1B3).

S2 Fig 1: uptake of 1 µM [^3^H]taurocholate in CHO-NTCP cells in function of incubation time. Values are expressed as mean ± SD (n = 3).

S2 Fig 2: uptake of 1 µM [^3^H]estradiol-17β-glucuronide in HEK-OATP1B1 cells in function of incubation time. Values are expressed as mean ± SD (n = 3).

S2 Fig 3: uptake of 1 µM [^3^H]estradiol-17β-glucuronide in HEK-OATP1B3 cells in function of incubation time. Values are expressed as mean ± SD (n = 3).
