# Supplementary material for: Synthesis, in vitro and in vivo evaluation of 3β-[18F]fluorocholic acid for the detection of drug-induced cholestasis in mice
Source: PLoS One. 2017 Mar 8;12(3):e0173529. doi: 10.1371/journal.pone.0173529 (PMC5342262; doi:10.1371/journal.pone.0173529)
Supplement: S3 File — (DOCX) [file pone.0173529.s003.docx]

S3 Table 1: Stability assessment of [^18^F]FCA in mouse serum and in presence of mouse primary hepatocytes.

|  | **[^18^F]FCA Recovery** | |
| --- | --- | --- |
| **Incubation time (min)** | **Mouse serum** | **Mouse primary hepatocytes** |
| 5 | 100 % | 100 % |
| 10 | 100 % | 100 % |
| 30 | 100 % | 100 % |
| 60 | 100 % | 100 % |

S3 Figure 1: Radiochromatogram of [^18^F]FCA in mouse serum: incubation for 5 minutes

S3 Figure 2: Radiochromatogram of [^18^F]FCA in mouse serum: incubation for 10 minutes

S3 Figure 3: Radiochromatogram of [^18^F]FCA in mouse serum: incubation for 30 minutes

S3 Figure 4: Radiochromatogram of [^18^F]FCA in mouse serum: incubation for 60 minutes

S3 Figure 5: Radiochromatogram of [^18^F]FCA in presence of primary mouse hepatocytes: incubation for 5 minutes

S3 Figure 6: Radiochromatogram of [^18^F]FCA in presence of primary mouse hepatocytes: incubation for 10 minutes

S3 Figure 7: Radiochromatogram of [^18^F]FCA in presence of primary mouse hepatocytes: incubation for 30 minutes

S3 Figure 8: Radiochromatogram of [^18^F]FCA in presence of primary mouse hepatocytes: incubation for 60 minutes
